# Prospect of potential intrauterine programming impacts associated with COVID-19

**DOI:** 10.3389/fpubh.2022.986162

**Published:** 2022-08-24

**Authors:** Prabhat Khanal, Asim K. Duttaroy

**Affiliations:** ^1^Faculty of Biosciences and Aquaculture, Nord University, Bodø, Norway; ^2^Department of Nutrition, Institute of Basic Medical Sciences, Faculty of Medicine, University of Oslo, Oslo, Norway

**Keywords:** COVID-19, fetal progamming, metabolic disease, nutrition, placenta

## Abstract

The severe acute respiratory syndrome coronavirus 2 (SARS-CoV-2) - 2019 (COVID-19) has led to a worldwide public health concern. In addition to immediate impacts on human health and well-being, COVID-19 can result in unfortunate and long-term health consequences for future generations. In particular, pregnant women and developing fetuses in low-income settings could be prone to a higher risk of undernutrition, often due to an inadequate supply of food and nutrition during a pandemic outbreak like COVID-19. Such situations can subsequently lead to an increased risk of undesirable health consequences, such as non-communicable diseases, including obesity, metabolic syndrome, hypertension, and type 2 diabetes, in individuals born to exposed mothers *via* fetal programming. Moreover, COVID-19 infection or related stress during pregnancy can induce long-term programming outcomes on neuroendocrinological systems in offspring after birth. However, the long-lasting consequences of the transplacental transmission of COVID-19 in offspring are currently unknown. Here we hypothesize that a COVID-19 pandemic triggers intrauterine programming outcomes in offspring due to multiple maternal factors (e.g., nutrition deficiency, stress, infection, inflammation) during pregnancy. Thus, it is crucial to establish an integrated lifetime health information system for individuals born in or around the COVID-19 pandemic to identify those at risk of adverse pre-and postnatal nutritional programming. This approach will assist in designing specific dietary or other nutritional interventions to minimize the potential undesirable outcomes in those nutritionally programmed individuals.

## Introduction

Coronavirus disease-2019 (COVID-19) represents a serious public health concern affecting people of all ages depending on geographical locations and socioeconomic conditions. As of 21st June 2022, over 500 million confirmed cases of COVID-19 have been reported globally, with more than 6 million deaths (1). Various preventive measures against COVID-19, such as lockdowns, limited physical distancing, self-isolation, etc., have been considered by governmental bodies across the globe to limit the transmission of SARS-CoV-2, the causative agent for COVID-19, to ensure that the capacity of healthcare systems would be able to combat the condition (2). COVID-19 leads to immediate impacts, such as fever, cough, fatigue, etc., on those who are infected (3) but also negative influences other patients that require urgent medical attention due to delayed access to the healthcare system for surgeries, chemotherapies, dialyses, etc. (4). In addition, multiple societal measures or interventions against pandemics like COVID-19 can also lead to undesirable consequences on people's metabolic health, exacerbating the prevalence of non-communicable diseases such as obesity, metabolic syndrome, hypertension, and type 2 diabetes (5). In this review, we highlight the potential role of COVID-19 on long-term intrauterine programming effects in relation to changes in food and nutrition supply, nutrition programming associated with specific nutrients, maternal stress, and transplacental SARS-CoV-2 transmission. Then, we generate a new hypothesis on COVID-19-associated programming outcomes of metabolic diseases in future generations.

## Methodology

A scoping review of specific scientific publications related to COVID-19 and fetal programming was conducted to evaluate the COVID-19-associated factors and their potential fetal programming impacts on long-term health and disease status. Databases such as Google Scholar, PubMed, Coronavirus resource center, etc., were used to search for peer-reviewed articles or specific COVID-19-related information. Different keywords and search terms, including “COVID-19” and “Maternal factors” and “Pregnancy” and “Fetal programming,” and “Placenta” were included as search terms.

## COVID-19 and nutrient supply

In addition to immediate adverse health impacts, COVID-19 has resulted in undesirable consequences on the global economy (6), consequently directly affecting the food and nutrition supply chains. The shortage of labor force, disturbances to existing transportation networks, and border closures affected the movement of food items across regions and countries (7), causing a temporary short supply of food. Moreover, panic buying and stockpiling of food products led to their pseudo-higher demands (8). Along with restrictions on the food supply, COVID-19 can have severe unfavorable consequences on global poverty. For example, it is estimated that COVID-19 can result in up to a 20 per cent contraction in household income or consumption, thus posing a severe threat to the UN Sustainable Development Goal of ending poverty by 2030 (9). Impacts of COVID-19 on the national economy and food security could be more prevalent in low-income countries (LICs) because of their history of sustained poverty and less resilient food supply chains. This can directly impact the nutritional status of vulnerable groups of people, particularly pregnant and lactating women, and children (10, 11). Furthermore, the inadequate nutrient supply (carbohydrates, protein, vitamin D, LCPUFAs, etc.), particularly for pregnant women, can have long-term consequences on the metabolic health of individuals born to those exposed mothers due to fetoplacental programming (12–14).

## The concept of nutritional programming

The concept of fetal or nutritional programming historically arose from several epidemiological studies conducted in countries from diverse regions. The studies reported that offspring born to mothers exposed to inadequate nutrient supply had higher risks of developing heart diseases (15, 16), impaired glucose tolerance, hypertension, and type 2 diabetes (17, 18). These studies led to the development of the “thrifty phenotype hypothesis” by Hales and Barker, strongly suggesting that poor nutrition during fetal and early postnatal life increases susceptibility toward the development of type 2 diabetes in adulthood (13). Additional studies in this field further pointed out that such long-term health consequences are associated with the nutritional status not only during fetal but also during the peri-conceptional or early postnatal period, leading to several conceptual frameworks, including the predictive adaptive responses (19), the developmental origin of health and diseases (DOHaD) (20). The hypotheses underlying these frameworks have been tested and confirmed in additional epidemiological studies, for example, using retrospective data from the Dutch famine (1944–1945) during the second world war (21, 22), and also in several animal studies, including but not limited to sheep (23–25), goats (26, 27), pigs (28–30), rodents (31–33), and monkeys (34, 35). These studies suggest that during unfortunate circumstances for humanity (e.g., during wars, natural disasters, or global emergencies like pandemic COVID-19), women during the peri-conceptional period or pregnancy can be exposed to mild to severe forms of undernutrition. This can subsequently lead to adverse health consequences and body functions in the offspring later in life.

## Nutritional programming associated with specific nutrients

The abnormal nutritional programming is associated with specific nutrient deficiencies during pregnancy. Choline, folic acid, Vitamin D, and long-chain polyunsaturated fatty acids (LCPUFAs) are among the nutrients that are important for alleviating maternal infection and inflammation in fetal growth and development (36). Pregnant women infected with SARS-CoV-2 can impart brain damage and post-birth psychiatric disorders in their offspring (37). SARS-CoV-2 can affect the development of the fetal nervous system directly or indirectly (38).

The maternal LCPUFAs and their metabolites are involved in every stage of pregnancy by supporting fetoplacental growth and development, cell signaling, and modulating other critical aspects of structural and functional processes (39). Inadequate trophoblast invasion of the maternal decidua and uterine spiral arterioles leads to structural and functional deficiency of the placenta, adversely affecting the overall fetal growth and the development of essential organs such as the brain (12, 40). During the third trimester of pregnancy, placental preferential transport of maternal plasma LCUPFAs is critical for fetal growth and development (12). DHA is essential for healthy brain development, maintenance, and function (12). DHA and its signaling systems are involved in neurogenesis, anti-nociceptive effects, anti-apoptotic effects, synaptic plasticity, and Ca^2+^ homeostasis in the brain. Studies strongly suggest that maternal dietary deficiency of DHA during pregnancy increases the risk for neurocognitive disorders. Maternal nutritional deficiency of n-3 fatty acids during development *in utero* and the postnatal state has detrimental effects on cognitive abilities (12). Vitamin D and folic acid are already supplemented in food additives and prenatal vitamins. Despite recommendations by several public health agencies and medical societies, choline intake is often inadequate in early gestation when the brain is forming. A public health initiative for choline supplements during the pandemic could be helpful for women planning or already pregnant who also become exposed or infected with SARS-CoV-2.

## Maternal stress and programming

COVID-19 has undoubtedly led to detrimental implications on the psychosocial welling and mental health status of people (41). It has been reported that COVID-19 increased stress and anxiety levels in pregnant women worldwide (42–44). Maternal stress can alter placental and fetal serotonin systems and expose the brain to increased corticotrophin-releasing hormone and cortisol, thus affecting fetal development and mental health status later in life (45, 46). In addition, maternal stress can modify the growth trajectory, locomotor activity, and adrenocortical responses to stress in offspring after birth (47). This suggests maternal stress during pregnancy due to pandemic situations such as COVID-19 can induce programming effects in developing fetuses affecting neuroendocrine systems and associated physiological body functions after birth.

## Intrauterine transplacental transmission of COVID-19

It is well known that COVID-19 is primarily transmitted through droplets and aerosols (48), although other modes of transmission may also prevail (49). In addition, the transplacental transmission of SARS-CoV-2 from a mother infected during late pregnancy to a neonate (neonatal viremia) has been reported where placental cells had a high viral load and showed inflammation under histological examination (50). Angiotensin-converting enzyme 2 (ACE-2) on the placental cell surface could play a role in the vertical transplacental transmission to the fetus following maternal COVID-19 infection (51). The long-term physiological impacts from such transmissions are currently unknown. However, it has been suggested SARS-CoV-2 infection in pregnancy may negatively influence fetal brain development *via* induction of maternal and placental immune activations (52).

## Conclusions

COVID-19 can lead to direct health impacts on infected pregnant women and result in unfortunate long-term health consequences for future generations. In particular, individuals born in low-income settings could be prone to inadequate maternal nutrient supply, maternal stress, and vertical transmission of SARS-CoV-2 from mothers. Such situations can subsequently lead to an increased risk of undesirable health consequences, including obesity, metabolic syndrome, hypertension, type 2 diabetes, and poor cognitive function in individuals born to exposed mothers *via* fetal programming.

## Future perspectives

**Figure 1:**
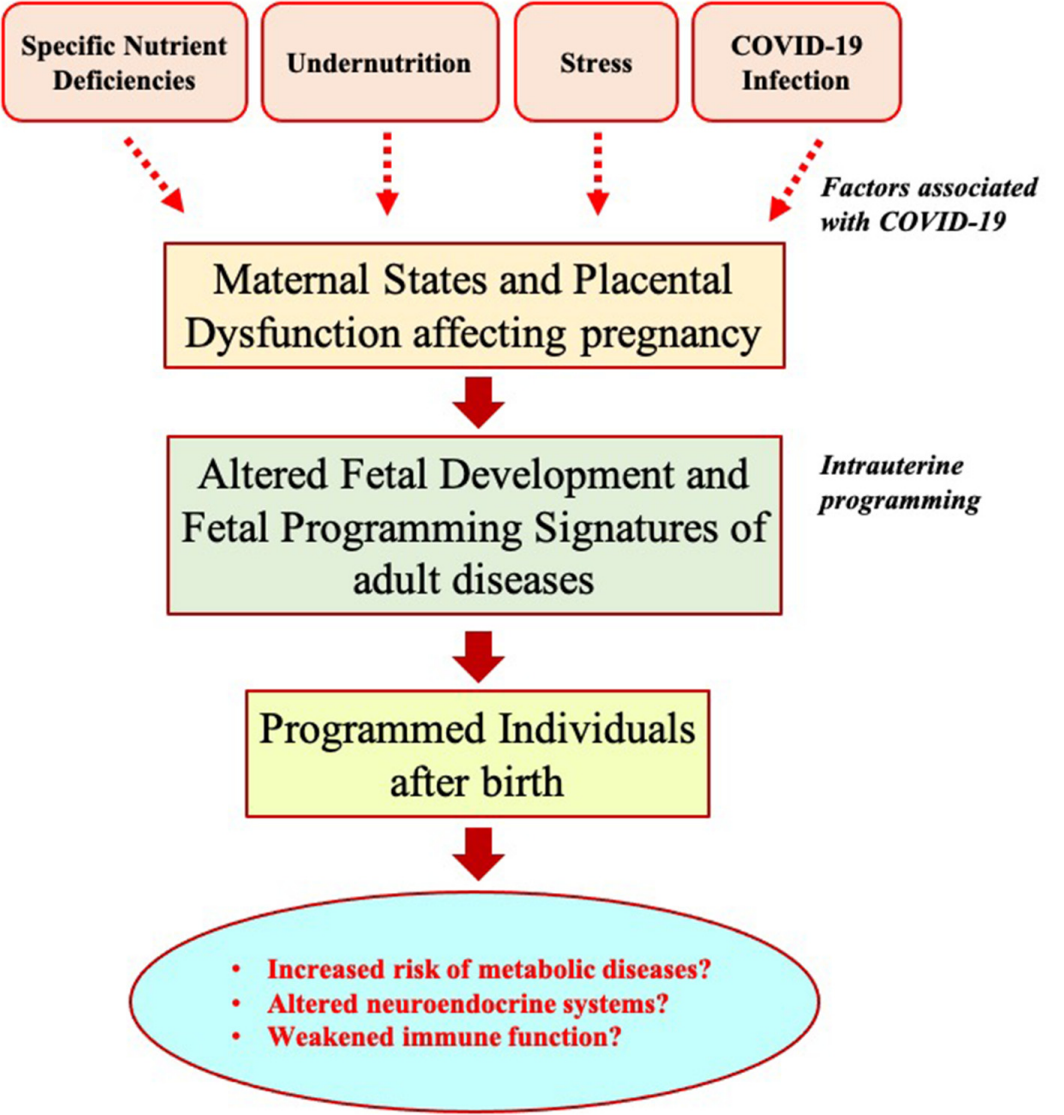
Potential influences of COVID-19 pandemic on long-term intrauterine programming outcomes.

COVID-19, one of the greatest public health crises in recent history, has suggested that it requires a global effort to address specific biological effects of SARS-CoV-2 infection in our future generations and design effective health policies for better preparedness and rapid response (53). Prenatal exposure to previous pandemics, such as the 1918 influenza pandemic or the Dutch famine (1944–1945), is found to be associated with increased risks of cardiovascular diseases in offspring later in life (54, 55). In this context, we hypothesize that children born during or immediately after this COVID-19 pandemic might have altered the development of fetal physiological systems *in utero* due to adverse intrauterine programming related to nutritional deficiency, stress, or infection during fetal life (Figure 1). COVID-19-associated abnormal nutritional programming could increase the risk of developing metabolic disorders such as hypertension, dyslipidemia, obesity, type-2 diabetes, and brain development in offspring after birth. Such outcomes of nutritional programming could be more harmful, particularly in the LICs, with a history of dealing with poverty and inequalities. To test the hypotheses, it is crucial to follow up the children in the future, particularly the LICs born during the post-COVID-19 period, by setting up longitudinal studies, such as prospective pregnancy/birth cohort studies, so that the potential increased risk of undesirable implications of intrauterine programming can be evaluated and relevant health strategies can be applied to minimize unfortunate health consequences. This requires careful management of the lifetime health data (53, 56), perhaps by developing properly integrated, scalable, and digitalized information systems, particularly in the health sector of the LICs (57). The use of robust data science and artificial intelligence approaches, particularly in the LICs, may provide immediate benefits toward a better response and recovery from a pandemic outbreak like COVID-19 and assist in identifying and following up on vulnerable groups that are at a higher risk of adverse nutritional programming. The e-health systems using modern information and communication technologies can also improve communication among health institutions, policymakers, and general communities (58). Such approaches are essential to follow up on susceptible individuals' dietary, physiological, and lifestyle behaviors and to design specific dietary, nutritional, or other health interventions to overcome the risks of undesirable and long-term consequences of outbreak-associated intrauterine programming in the future. Furthermore, future research should focus on epigenetic programming to help to understand potential mechanisms and timing of exposures for long-term adverse health effects of COVID-19 and future protracted human emergencies.

## Author contributions

PK: conceptualization, writing, revision, and approval of the final version. AD: writing, revision, and approval of the final version. Both authors contributed to the article and approved the submitted version.

## Conflict of interest

The authors declare that the research was conducted in the absence of any commercial or financial relationships that could be construed as a potential conflict of interest.

## Publisher's note

All claims expressed in this article are solely those of the authors and do not necessarily represent those of their affiliated organizations, or those of the publisher, the editors and the reviewers. Any product that may be evaluated in this article, or claim that may be made by its manufacturer, is not guaranteed or endorsed by the publisher.

## References

[1] John Hopkins University. John Hopkins University, Coronavirus Resource Center. Available online at: https://coronavirus.jhu.edu/map.html (accessed June 21, 2022).

[2] Anderson RM Heesterbeek H Klinkenberg D Hollingsworth TD. How will country-based mitigation measures influence the course of the COVID-19 epidemic? Lancet. (2020) 395:931–4. 10.1016/S0140-6736(20)30567-5 32164834 PMC7158572

[3] Alimohamadi Y Sepandi M Taghdir M Hosamirudsari H. Determine the most common clinical symptoms in COVID-19 patients: a systematic review and meta-analysis. J Prev Med Hyg. (2020) 61:E304. 10.15167/2421-4248/jpmh2020.61.3.1530 33150219 PMC7595075

[4] Riera R Bagattini ÂM Pacheco RL Pachito DV Roitberg F Ilbawi A. Delays and disruptions in cancer health care due to COVID-19 pandemic: systematic review. JCO Global Oncol. (2021) 7:311–23. 10.1200/GO.20.00639 33617304 PMC8081532

[5] Clemmensen C Petersen MB Sørensen TI. Will the COVID-19 pandemic worsen the obesity epidemic? Nat Rev Endocrinol. (2020) 16:469–70. 10.1038/s41574-020-0387-z 32641837 PMC7342551

[6] McKibbin WJ Fernando R. The global macroeconomic impacts of COVID-19: Seven scenarios CAMA Working Paper No 19/. (2020). 10.2139/ssrn.3547729

[7] Hobbs JE. Food supply chains during the COVID-19 pandemic. Can J Agric Econ. (2020) 68:171–6. 10.1111/cjag.1223734908599

[8] Nicola M Alsafi Z Sohrabi C Kerwan A Al-Jabir A Iosifidis C. The socio-economic implications of the coronavirus pandemic (COVID-19): a review. Int J Surg. (2020) 78:185–93. 10.1016/j.ijsu.2020.04.018 32305533 PMC7162753

[9] Sumner A Hoy C Ortiz-Juarez E. Estimates of the Impact of COVID-19 on Global Poverty. Helsinki: UNU-WIDER, April, (2020), 800–809. 10.35188/UNU-WIDER/2020/800-9

[10] Dunn CG Kenney E Fleischhacker SE Bleich SN. Feeding low-income children during the Covid-19 pandemic. N Engl J Med. (2020) 382:e40. 10.1056/NEJMp2005638 32227759

[11] Pérez-Escamilla R Cunningham K Moran VH. COVID-19, food and nutrition insecurity and the wellbeing of children, pregnant and lactating women: a complex syndemic. Matern Child Nutr. (2020) 16:e13036. 10.1111/mcn.13036 32458574 PMC7267083

[12] Basak S Mallick R Duttaroy AK. Maternal docosahexaenoic acid status during pregnancy and its impact on infant neurodevelopment. Nutrients. (2020) 12:3615. 10.3390/nu12123615 33255561 PMC7759779

[13] Hales CN Barker DJ. Type 2 (non-insulin-dependent) diabetes mellitus: the thrifty phenotype hypothesis. Diabetologia. (1992) 35:595–601. 10.1007/BF00400248 1644236

[14] Lucas A. Programming by early nutrition in man. The childhood environment and adult disease. Ciba Found Symp. (1991) 1991:38–55. 10.1002/9780470514047.ch4 1855415

[15] Barker DJ Osmond C Winter P Margetts B Simmonds SJ. Weight in infancy and death from ischaemic heart disease. Lancet. (1989) 334:577–80. 10.1016/S0140-6736(89)90710-1 2570282

[16] Forsdahl A. Are poor living conditions in childhood and adolescence an important risk factor for arteriosclerotic heart disease? J Epidemiol Community Health. (1977) 31:91–5. 10.1136/jech.31.2.91 884401 PMC479002

[17] Barker DJ Bull AR Osmond C Simmonds SJ. Fetal and placental size and risk of hypertension in adult life. Br Med J. (1990) 301:259–62. 10.1136/bmj.301.6746.259 2390618 PMC1663477

[18] Hales CN Barker DJ Clark PM Cox LJ Fall C Osmond C. Fetal and infant growth and impaired glucose tolerance at age 64. BMJ. (1991) 303:1019–22. 10.1136/bmj.303.6809.1019 1954451 PMC1671766

[19] Gluckman PD Hanson MA Spencer HG. Predictive adaptive responses and human evolution. Trends Ecol Evol. (2005) 20:527–33. 10.1016/j.tree.2005.08.001 16701430

[20] Gluckman PD Hanson MA. The developmental origins of health and disease. In: Early Life Origins of Health And Disease. Berlin: Springer (2006), p. 1–7. 10.1017/CBO9780511544699

[21] Ravelli AC van der Meulen JH Michels R Osmond C Barker DJ Hales C. Glucose tolerance in adults after prenatal exposure to famine. Lancet. (1998) 351:173–7. 10.1016/S0140-6736(97)07244-9 9449872

[22] Roseboom TJ van der Meulen JH Osmond C Barker DJ Ravelli AC Schroeder-Tanka JM. Coronary heart disease after prenatal exposure to the Dutch famine, 1944–45. Heart. (2000) 84:595–8. 10.1136/heart.84.6.595 11083734 PMC1729504

[23] Khanal P Husted SV Axel AMD Johnsen L Pedersen KL Mortensen MS. Late gestation over-and undernutrition predispose for visceral adiposity in response to a post-natal obesogenic diet, but with differential impacts on glucose–insulin adaptations during fasting in lambs. Acta Physiol. (2014) 210:110–126. 10.1111/apha.12129 23746217

[24] Khanal P Pandey D Binti Ahmad S Safayi S Kadarmideen HN Olaf Nielsen M. Differential impacts of late gestational over–and undernutrition on adipose tissue traits and associated visceral obesity risk upon exposure to a postnatal high-fat diet in adolescent sheep. Physiol Rep. (2020) 8:e14359. 10.14814/phy2.14359 32026612 PMC7002533

[25] Symonds ME Stephenson T Gardner DS Budge H. Long-term effects of nutritional programming of the embryo and fetus: mechanisms and critical windows. Reprod Fertil Dev. (2006) 19:53–63. 10.1071/RD06130 17389135

[26] He Z Wu D Sun Z Tan Z Qiao J Ran T. Protein or energy restriction during late gestation alters fetal growth and visceral organ mass: an evidence of intrauterine programming in goats. Anim Reprod Sci. (2013) 137:177–82. 10.1016/j.anireprosci.2013.01.005 23395360

[27] Yang C Zhou X Yang H Gebeyew K Yan Q Zhou C. Transcriptome analysis reveals liver metabolism programming in kids from nutritional restricted goats during mid-gestation. PeerJ. (2021) 9:e10593. 10.7717/peerj.10593 33575124 PMC7849524

[28] Bauer R Walter B Bauer K Klupsch R Patt S Zwiener U. Intrauterine growth restriction reduces nephron number and renal excretory function in newborn piglets 1. Acta Physiol Scand. (2002) 176:83–90. 10.1046/j.1365-201X.2002.01027.x 12354166

[29] Cao M Che L Wang J Yang M Su G Fang Z. Effects of maternal over-and undernutrition on intestinal morphology, enzyme activity, and gene expression of nutrient transporters in newborn and weaned pigs. Nutrition. (2014) 30:1442–7. 10.1016/j.nut.2014.04.016 25280425

[30] Wang J Cao M Zhuo Y Che L Fang Z Xu S. Catch-up growth following food restriction exacerbates adulthood glucose intolerance in pigs exposed to intrauterine undernutrition. Nutrition. (2016) 32:1275–84. 10.1016/j.nut.2016.03.010 27210508

[31] Kozak LP Newman S Chao P-M Mendoza T Koza RA. The early nutritional environment of mice determines the capacity for adipose tissue expansion by modulating genes of caveolae structure. PLoS ONE. (2010) 5:e11015. 10.1371/journal.pone.0011015 20574519 PMC2888576

[32] Langley-Evans SC. Fetal programming of cardiovascular function through exposure to maternal undernutrition. Proc Nutr Soc. (2001) 60:505–13. 10.1079/PNS2001111 12069404

[33] Watkins AJ Lucas ES Wilkins A Cagampang FR Fleming TP. Maternal periconceptional and gestational low protein diet affects mouse offspring growth, cardiovascular and adipose phenotype at 1 year of age. PLoS ONE. (2011) 6. 10.1371/journal.pone.0028745 22194901 PMC3240629

[34] Clarke AS Wittwer D Abbott D Schneider M. Long-term effects of prenatal stress on HPA axis activity in juvenile rhesus monkeys. Dev Psychobiol. (1994) 27:257–69. 10.1002/dev.420270502 7926279

[35] Hinde K Capitanio JP. Lactational programming? Mother's milk energy predicts infant behavior and temperament in rhesus macaques (*Macaca mulatta*). Am J Primatol. (2010) 72:522–9. 10.1002/ajp.20806 20162547 PMC3377500

[36] Beluska-Turkan K Korczak R Hartell B Moskal K Maukonen J Alexander DE. Nutritional gaps and supplementation in the first 1000 days. Nutrients. (2019) 11:2891. 10.3390/nu11122891 31783636 PMC6949907

[37] López-Díaz Á Ayesa-Arriola R Crespo-Facorro B Ruiz-Veguilla M. COVID-19 infection during pregnancy and risk of neurodevelopmental disorders in offspring: time for collaborative research. Biol Psychiatry. (2021) 89:e29–30. 10.1016/j.biopsych.2020.09.011 33131716 PMC7604160

[38] Wang R Wu Z Huang C Hashimoto K Yang L Yang C. Deleterious effects of nervous system in the offspring following maternal SARS-CoV-2 infection during the COVID-19 pandemic. Transl Psychiatry. (2022) 12:1–6. 10.1038/s41398-022-01985-z 35668063 PMC9169439

[39] Basak S Mallick R Banerjee A Pathak S Duttaroy AK. Maternal supply of both arachidonic and docosahexaenoic acids is required for optimal neurodevelopment. Nutrients. (2021) 13:2061. 10.3390/nu13062061 34208549 PMC8234848

[40] Basak S Duttaroy AK. Maternal PUFAs, placental epigenetics, and their relevance to fetal growth and brain development. Reprod Sci. (2022). 10.1007/s43032-022-00989-w [Epub ahead of print]. 35676498

[41] Pfefferbaum B North CS. Mental health and the Covid-19 pandemic. N Engl J Med. (2020) 383:510–2. 10.1056/NEJMp2008017 32283003

[42] Effati-Daryani F Zarei S Mohammadi A Hemmati E Ghasemi Yngyknd S Mirghafourvand M. Depression, stress, anxiety and their predictors in Iranian pregnant women during the outbreak of COVID-19. BMC Psychol. (2020) 8:1–10. 10.1186/s40359-020-00464-8 32962764 PMC7506842

[43] Medina-Jimenez V Bermudez-Rojas ML Murillo-Bargas H Rivera-Camarillo AC Muñoz-Acosta J Ramirez-Abarca TG. The impact of the COVID-19 pandemic on depression and stress levels in pregnant women: a national survey during the COVID-19 pandemic in Mexico. J Matern Fetal Neonatal Med. (2020). 10.1080/14767058.2020.1851675 [Epub ahead of print]. 33243043

[44] Preis H Mahaffey B Heiselman C Lobel M. Vulnerability and resilience to pandemic-related stress among US women pregnant at the start of the COVID-19 pandemic. Soc Sci Med. (2020) 266:113348. 10.1016/j.socscimed.2020.113348 32927382 PMC7474815

[45] St-Pierre J Laurent L King S Vaillancourt C. Effects of prenatal maternal stress on serotonin and fetal development. Placenta. (2016) 48:S66–71. 10.1016/j.placenta.2015.11.013 26691753

[46] Weinstock M. The potential influence of maternal stress hormones on development and mental health of the offspring. Brain Behav Immun. (2005) 19:296–308. 10.1016/j.bbi.2004.09.006 15944068

[47] Emack J Kostaki A Walker C-D Matthews SG. Chronic maternal stress affects growth, behaviour and hypothalamo–pituitary–adrenal function in juvenile offspring. Horm Behav. (2008) 54:514–20. 10.1016/j.yhbeh.2008.02.025 18674758

[48] Jayaweera M Perera H Gunawardana B Manatunge J. Transmission of COVID-19 virus by droplets and aerosols: a critical review on the unresolved dichotomy. Environ Res. (2020) 188:109819. 10.1016/j.envres.2020.109819 32569870 PMC7293495

[49] Galbadage T Peterson BM Gunasekera RS. Does COVID-19 spread through droplets alone? Front Public Health. (2020) 8:163. 10.3389/fpubh.2020.00163 32391310 PMC7193306

[50] Vivanti AJ Vauloup-Fellous C Prevot S Zupan V Suffee C Do Cao J. Transplacental transmission of SARS-CoV-2 infection. Nat Commun. (2020) 11:1–7. 10.1038/s41467-020-17436-6 32665677 PMC7360599

[51] Wong YP Khong TY Tan GC. The effects of COVID-19 on placenta and pregnancy: what do we know so far? Diagnostics. (2021) 11:94. 10.3390/diagnostics11010094 33435547 PMC7827584

[52] Shook LL Sullivan EL Lo JO Perlis RH Edlow AG. COVID-19 in pregnancy: implications for fetal brain development. Trends Mol Med. (2022). 10.1016/j.molmed.2022.02.004 PMC8841149 35277325

[53] Roseboom TJ Ozanne SE Godfrey KM Isasi CR Itoh H Simmons R. Unheard, unseen and unprotected: DOHaD council's call for action to protect the younger generation from the long-term effects of COVID-19. J Dev Orig Health Dis. (2021) 12:3–5. 10.1017/S2040174420000847 32962780

[54] Mazumder B Almond D Park K Crimmins EM Finch CE. Lingering prenatal effects of the 1918 influenza pandemic on cardiovascular disease. J Dev Orig Health Dis. (2010) 1:26–34. 10.1017/S2040174409990031 20198106 PMC2826837

[55] Roseboom T de Rooij S Painter R. The Dutch famine and its long-term consequences for adult health. Early Hum Dev. (2006) 82:485–91. 10.1016/j.earlhumdev.2006.07.001 16876341

[56] Khanal P Nielsen MO. Is foetal programming by mismatched pre-and postnatal nutrition contributing to the prevalence of obesity in Nepal? Prev Nutr Food Sci. (2019) 24:235. 10.3746/pnf.2019.24.3.235 31608248 PMC6779080

[57] Braa J Hanseth O Heywood A Mohammed W Shaw V. Developing health information systems in developing countries: the flexible standards strategy. MIS Q. (2007) 381–402. 10.2307/25148796

[58] Blaya JA Fraser HS Holt B. E-health technologies show promise in developing countries. Health Aff. (2010) 29:244–51. 10.1377/hlthaff.2009.0894 20348068

